# Preoxygenation and Anesthesia: A Detailed Review

**DOI:** 10.7759/cureus.13240

**Published:** 2021-02-09

**Authors:** Mohammed Azam Danish

**Affiliations:** 1 Anesthesiology, Bhagwan Mahaveer Jain Hospital, Bangalore, IND

**Keywords:** anesthesia, intubation, preoxygenation, risk factors, apnea

## Abstract

Initiation of preoxygenation prior to anesthetic induction and tracheal intubation is a commonly recognized technique intended to boost oxygen reservoirs in the body and thus slow the progression of desaturation of arterial hemoglobin at times of apnea. Even though challenges associated with ventilation and intubation are inconsistent, it is preferable for all patients to necessitate preoxygenation. The effectiveness of preoxygenation is measured by its performance and efficiency. Determinant factors of efficacy indices include rises in the alveolar O2 fraction (FAO2), reductions in the alveolar nitrogen fraction (FAN2), and improvements in the arterial O2 stress (PAO2). The effectiveness or efficiency of preoxygenation during apnea is evaluated from the declining trend in level of oxyhemoglobin desaturation (SAO2). The maximal risk associated with preoxygenation generally comprises delayed diagnosis of oesophageal intubation, absorption atelectasis, generation of reactive oxygen species, and incidences of adverse hemodynamic results. Since the time of preoxygenation is minimal, there are limited hemodynamic effects and the aggregation of reactive oxygen species to counteract its effectiveness. In general, three methods of preoxygenation techniques are followed for the routine procedures, namely, deep breathing, rapid breathing at fraction of inspired oxygen (FiO2) of 1 for two to five minutes, and the four vital capacities method. Health professionals, especially anesthesiologists specialized in Ear Nose and Throat (ENT) and traumatology, must be empowered by alternative methods like trans-tracheal ventilation to resolve life-threatening medical emergencies. Equipment accessibility and needful training are two essential components that are recommended for significant preparedness. The present article reviews the advantages conferred by the preoxygenation techniques with special attention to the high-risk population. It also details the inadequacies and the risks associated with the preoxygenation technique.

## Introduction and background

The primary objective of preoxygenating a patient to the maximum extent prior to the induction of general anesthesia and paralysis is to offer the maximum amount of time a patient can withstand apnea, and to help address a non-ventilate, non-intubate situation for the anesthesia provider [1]. Maximum preoxygenation is attained when alveolar, arterial, tissue, and venous compartments are oxygen-filled. Consequently, patients with either reduced oxygen loading capacity or enhanced oxygen extraction, or both, desaturate during apnea much faster than a healthy one. Preoxygenation ensures a comfortable shield during times of apnea and hypoventilation [2]. It broadens the safe apnea time period, described as the duration until a patient attains a saturation limit of 88% to 90%, thereby enabling the precise airway. If patients are desaturated below this point, their state stands on the steep portion of oxyhemoglobin dissociation curve and can drop to critical oxygen saturation levels within minutes [3]. The conventional induction of anesthesia in operative patients is executed by the administration of a sedative, with manual ventilation and administration of a muscle relaxant with continuation of manual ventilation before a definite airway is positioned. Since they are physiologically normal with low metabolic requirements, preoxygenation in these cases is not necessary, since ventilation persists throughout the induction period. For surgical patients with a significant risk of aspiration due to serious illness, anesthesiologists devised rapid sequence induction. As this strategy was originally developed and consists of sequential sedative and paralytic treatment without ventilation relying on the paralytic to come into force, unless needed to prevent hypoxemia. This method of induction was tailored to use in the Emergency Department (ED) where all patients who need control of the airways are considered to be at risk of aspiration, the default methodology is rapid sequence tracheal intubation [4].

There arise three major objectives to be fulfilled in the ED, comprising bringing patient saturation as near as possible to 100%, denitrogenating the residual lung capacity or maximizing the storage of oxygen in the lungs, and denitrogenation and oxygenating the bloodstream to a maximal level [5]. Blood denitrogenisation and oxygenation leads to safe apnea for a limited period, since oxygen is weakly soluble in blood, with a relatively low reservoir of oxygen relative to the lungs [6].

Patients should preferably appear to experience preoxygenation until they denitrogenate their lungs' residual capacity adequate to reach end-tidal oxygen above 90%. For the majority of patients, breathing in three minutes of tidal-volume with an elevated source of oxygen (FiO2) is an adequate preoxygenation time. This tidal volume respiration method can be improved by advising the patient for maximal exhalation followed by maximal inhalation before the three-minute time mark [7].

Preoxygenation prior to anesthesia is particularly required when mask ventilation becomes difficult to handle. Such instances arise in patients who are prone to fast desaturation, like those who are overweight, pregnant or febrile or associated with pulmonary diseases, patients with assumed full abdomen; when mask ventilation problems are predicted, at times when tracheal intubation could take longer than anticipated time; in cases where special intubation techniques for managing airways are needful such as placement of a double lumen tube. Because unforeseen tracheal intubation difficulties are fairly common, all patients are recommended with preoxygenation before general anaesthetic induction [8].

The present article reviews the advantages and preoxygenation techniques with special attention to the high-risk population as well. Concerns have been expressed in the literature over the decades in relation to the potential adverse effects associated with preoxygenation. Such results include delayed detection of esophagus intubation, propensity to induce absorption atelectasis, and development of reactive oxygen species, as well as hemodynamic adverse changes.

## Review

Reasons for preoxygenation inadequacies

Patient-Related Factors

It could be hard or impossible to preoxygenate if the patient lacks cooperation, is claustrophobic or particularly anxious, and in a condition where anesthesia needs to be administered very fast. In such scenarios, the faster methodologies could be useful as they need less time and it is presumed that a patient could comply better if they are asked to execute a short-term task, which necessitates some consideration and focus [9].

Technical Factors

Even though fraction of expired oxygen (FeO2) can be attained 95% theoretically, reaching 90% FeO2 in practical terms is generally considered acceptable. The major reasons to attain the above-said values may be attributed either to a lower flow of oxygen, presence of leaks, or insufficient time period of preoxygenation. Among the above-mentioned factors, detection of leaks remains difficult to address and it has been reported that approximately 11.5% of the patients with edentulous or bearded subjects or in subjects with face anomalies, burns, or the presence of a nasogastric tube, face this problem [10,11].

Positioning of patient to receive preoxygenation

Supine placement is not considered to be suitable for optimal results. It becomes hard to start taking full breaths when placed flat and more of the rear lung gets prone to atelectatic collapse. It leads to a drop in the oxygen reservoir contained within the lungs and thereby lowers safe apnea time consequently. A number of reports have been published in relation to the proper positioning of the patient to receive preoxygenation. A randomized controlled trial was conducted in patients who were preoxygenated in a 20-degree head-up position vs. a control group of patients who were maintained in a supine position. After a period of three minutes, the patients received sedation and muscle relaxants. The patients were then allowed to reduce their saturation levels to 95% from 100%. The patients in the control group took 283 seconds to reach the level while the ones in the head-up group took 386 seconds [12]. A similar report was also published by Ramkumar et al. [13].

Hence, it may be anticipated that patients should be preoxygenated in the head-up position. Reverse Trendelenburg may be indicated in immobilized patients with a spinal injury.

Preoxygenation techniques

In situations where there is a possible risk of desaturation before the use of endotracheal intubation to protect the airways, pre-oxygenation is highly encouraged during anesthesia induction. During its absence, there is an increasing likelihood of desaturation. The device needs to be customized and configured securely to the patient, specifically the face mask. An anatomical discrepancy between the mask and the patient's face such as inadequate mask size, beards, or mustaches prohibits the sealing perfectly and may lead to failure [14]. The mask must be firmly adapted to the patient's face. It is a known fact that 20% dilution of O2 by surrounding air tends to occur when the mask is not placed firmly, and O2 dilution of 40% arises when it is moved closer to the face [15]. The fresh gas flow circle system encompassing a flow rate of 5 L/min is employed as a benchmark for comparison in anesthesia experiments analyzing the effect of various circuits [16]. Until preoxygenation, O2 needs to be pumped in to the circuit and reservoir. Broadly four methods of preoxygenation are employed for the routine procedures, namely deep breathing, rapid breathing at fraction of inspired oxygen (FiO2) of 1 for two to five minutes, four vital capacities method and Transnasal Humidified Rapid Insufflation Ventilator Exchange (THRIVE).

Deep Breathing

A convenient approach of preoxygenation is eight deep breaths at an oxygen flow rate of 10 L/min within a time period of 60 seconds [17]. This procedure resulted in an average arterial oxygen tension of 369±69 mmHg that is not substantially distinct from the level obtained by three minutes of tidal volume breathing at 5 L/min of oxygen flow [17].

Rapid Breathing at FiO2

This specific method produces denitrogenation with an alveolar O2 fraction (FAO2) of approximately 95% in patients with pre-existing normal pulmonary function. Denitrogenation is beneficial from the first minute when preoxygenation is initiated, however, circuit leakage nullifies the above-stated effects through a rapid reduction in FiO2 [18]. Pure O2 breathing for extended time periods of more than a minute seems to be of very less significant advantages of oxygen saturation (SpO2) or alveolar denitrogenation, but significantly improves apnea period prior to arterial desaturation [19]. In studies with healthy volunteers, apnea time that is sustained until the SpO2 reaches more than 90%, can be prolonged to nearly 10 minutes after a time point of three minutes of standard pre-oxygenation technique. Applying positive pressure all through preoxygenation and ventilation in the mask post-induction can significantly raise the time of apnea by an extra added two minutes [20].

Four Vital Capacities Method

The four vital capacities method is implemented in situations where patient cooperation is compromised. Following four power manoeuvres, the length of apnea without desaturation is shortened as compared to spontaneous breathing. The restrictions of this technique are accountable for practical prerequisites such as bag capacity, inspiratory flow, and room gas inspiration. The vital capacity maneuver procedure ideally continues with such a forced expiration to maximize the elevation of FeO2 [21]. In order to be completely successful, the inspiratory O2 flow should be higher than the maximum inspiratory flow obtained by triggering the by-pass O2 mechanism while inspiration; four or five forced mere O2 breathing have been reported to be as productive as conventional pre-oxygenation evaluated on FeO2 [22].

Transnasal Humidified Rapid Insufflation Ventilator Exchange (THRIVE)

This methodology is followed when a difficult airway in patients is forecast. During the stated procedure, oxygen is maintained at a flow rate of 70 L/min and maintained till induction and post-administration of neuromuscular blockade to provide apnoeic oxygenation.

Preoxygenation in high-risk patients

Aged/Elderly Patients

Aging in humans comes up with major structural and physiological attributes in the respiratory system, which predominantly comprises of weak muscles of the respiratory system with parenchymal changes in the lungs, related to reduced elasticity [23]. Basal oxygen uptake (VO2) drops with aging, disordered intake of O2 results in faster desaturation all through apnea under anaesthesia. Tidal volume breathing for a time period of three minutes or longer was marked to be more efficient than four deep respiratory techniques in elderly patients [24].

Pediatric Patients

Reports have shown that maximum preoxygenation can be obtained in children (ETO2 = 90 percent) and is generally achieved faster than in adults. A 90% ETO2 is achieved in the majority of children with tidal volume breathing within 100 seconds, while it can be attained in 30 seconds with deep respiration [25]. Children generally face an increased chance of developing hypoxemia in conditions when delivery of O2 is disrupted, such as during apnea or an obstruction to the airway as they tend to have a shorter residual capacity and a higher VO2 as compared to adults. Preoxygenation contributes more for an older child. It is reported that the time frame of a secure period of apnea for an eight-year-old child can be broadened to five minutes or longer with preoxygenation from 0.47 minutes with no preoxygenation. The onset of desaturation becomes fast in low-age-group children [26].

Pregnant Patients

Most often, intubation is carried out in pregnant women who are induced with general anesthesia and preoxygenation is termed to be essential in these patients. Overall preoxygenation is possible faster owing to a pregnancy higher than in non-pregnant women due to incidence of less residual functional capacity and alveolar ventilation [27]. Consequently, throughout apnea, pregnant women continue to build desaturation of oxyhemoglobin faster since the minimal amount of O2 in their smaller residual capacity coupled with an improved VO2. It has been reported elsewhere that a head-up position of 45° leads to an enhancement of the desaturation time more in non-pregnant women than in pregnant ones. The reason for the observed effect may be attributed to the fact that the uterus may have been responsible for preventing the exit of the diaphragm and does not allow the increase expected efficient residual capacity at head-up position [28]. It is proposed that the four deep breathing techniques are inferior in pregnant women than the three-minute tidal volume technique and need not be advocated except in emergencies. Hence, it is recommended that enhanced ventilation in the said categories a minute longer for pregnant women for ventilation demands an O2 supply of 10 L/min during the time of preoxygenation [29].

Patients With Pulmonary Complications

The effectiveness and performance of both can be adversely affected by the prevalence of associated lung disease. Severe pulmonary disease is often related to a reduced residual functional capacity, significant perfusion malfunction- ventilation along with an increased VO2, that can lower the safety margin. Patients with chronic obstructive pulmonary disease are often seen to be associated with impairment to gas exchange after anesthesia induction. Brief ventilation disturbance such as when suctioning often results in substantial desaturation in these categories of patients [30].

Obese Patients

Research has shown after preoxygenation procedure, breathing accompanied with tidal volume for a period of three minutes, the time needed for SaO2 to reduce to 90% during apnea dramatically gets dropped in obese patients with a calculated BMI >40 kg/m2 than in non-obese patients [31]. After preoxygenation during apnea, the average time period to hit a SaO2 of 90% was 2.7 minutes in obese patients, while in patients with normal body weight it lasted for six minutes. Prevalence of obstructive apnea of sleep is often seen in obese patients. This leads to conditions in patients, which makes mask ventilate and intubation difficult to manage. Desaturation in oxyhemoglobin levels during apnea is directly linked to obese patients with higher VO2 and a significantly decreased functional residual capacity. Severely obese patients placed in head-up position of 25° have demonstrated to extend the time of desaturation during preoxygenation by about a time period of 50 seconds [32]. Some anesthesiologists might recommend awake fibreoptics intubation instead of rapid sequence intubation in morbidly obese patients with a calculated BMI >50kg/m2. Associated advantage of this strategy is airway patency maintenance during normal process of breathing [33].

Risk associated with preoxygenation techniques

The various risk associated with preoxygenation techniques are detailed below:

Absorption Atelectasis

Atelectasis is the most common side effect associated with preoxygenation and is reported to occur in approximately 75 to 90% of people who undergo general anesthesia [34]. The precipitation of absorption atelectasis occurs by two major mechanisms. The first mechanism involves complete occlusion of the airways, producing a reservoir of trapped gas within the distal lung structure. At first, the pressure in the reservoir is near atmospheric pressure. Mixed venous blood persists to perfuse the reservoir. As the sum total of the partial pressure of gas in the mixed venous blood is subatmospheric, the blood starts to absorb gas from the reservoir resulting in its collapse [35]. The second mechanism by which absorption atelectasis occurs is when the inspired ratio of ventilation/perfusion (VA/Q) is less than critical VA/Q ratio. This then leads to collapse of lung unit [36].

The methodologies that can be followed to reduce absorption atelectasis following preoxygenation include the use of recruitment maneuvers and reducing FiO2 concentration [37].

Cardiovascular Disorders

During preoxygenation, cardiovascular responses have been given marginal attention and were not well represented. Yet there are a number of research studies conducted both in humans and animals. Cardiovascular steady-state evaluation during high O2 breathing was studied which can provide a wider perspective into the variations in hemodynamic conditions during preoxygenation. Several experiments have been performed on normal male subjects which concluded that 100% O2 respiration generates a moderate decline in heart rate complemented by a simultaneous reduction in heart output. This leads to a rise in systemic blood pressure and arterial blood pressure [38]. These improvements are a result of reflex loop, which may be chemoreceptor or baroreceptor in origin. The effect of 100% O2 inhalation was evaluated in a variety of physiological studies [39]. Hyperoxia was reliably responsible for a pronounced decline in coronary blood flow along with a drop in myocardial oxygen distribution. The significant effect of coronary vasoconstrictors of hyperoxia is a result of the oxidative inactivation of nitric oxide as well as other activated vasodilators released from endothelium and the closure of K+ channels [40]. Experiments in normal coronary artery patients have shown that, irrespective of the decline in coronary blood circulation, oxygenation at myocyte level stays sufficient, as demonstrated by continued myocardial lactate extraction [41]. It is possibly explained by an improved capacity of the arterial O2 production to blunt down the supply of coronary O2 supply triggered by a cumulative decreased coronary blood flow along with a reduced demand for myocardial O2, relative to bradycardia caused by hyperoxia. Metabolic insights in severe coronary artery disease patients have been noted to be incongruous. Studies in animal models have shown that hyperoxia remains a responsible factor for causing vasoconstriction and a reduction in blood flow in peripheral artery beds like the kidney, GI tract, and hind limb [42]. The vasoconstriction may be attributed to the significant impact of O2 on vascular smooth muscle or reflex guided by an arterial chemoreceptor/nerve autonomy, which still remains an open question.

Generation of Reactive Oxygen Species

The dioxygen molecule in biological tissues could be split inadvertently creating reactive species of oxygen-containing superoxide anion, hydroxyl radical, and hydrogen peroxide [43]. The produced reactive oxygen species can interact with biological components such as lipids, DNA, and proteins, which can result in significant cellular damage [44]. However even if the presence of endogenous antioxidants are routinely enough to avoid high tissue concentrations of the accumulated reactive oxygen species, the associated mechanisms can sometimes become overpowered which induces oxidative stress. Specific clinical conditions that result in an increase in production of reactive oxygen species are pulmonary edema, acute respiratory distress syndrome, retinal detachment, retinopathy of prematurity, and seizures [45].

## Conclusions

Preoxygenation preceding anesthetic induction and intubation by trachea is a broadly acknowledged strategy aimed at increasing oxygen storage in the body and thus, postpones the development of arterial hemoglobin desaturation during the process of apnea. Earlier published literature provides conclusive evidence of the fact that preoxygenation implemented before or after induction delays the emergence of hypoxemia during apnea. Efficient preoxygenation FeO2 >90% is crucial during the process of airway management to prevent hypoxemia. Some circumstances during induction present a higher risk factor such as pregnancy, obesity, rapid sequence of induction, Hence, entail distinctive attention. These at-risk scenarios can be foreseen by identifying potential risks. Preoxygenation needs to be conducted whenever predicted obstruction of delivery of O2 is evidenced such as during open tracheobronchial suction and before and during awake fiberoptic intubation. Hence, it can be inferred that preoxygenation needs to be performed in all patients who are given general anesthesia. For most cases, supplementary O2 enhances the time duration of manageable apnea following pre-oxygenation, and this very basic step should not be ignored. Pre-oxygenation deficiencies must be outlined, and appropriate oxygenation techniques should be readily accessible for faster and easier execution. To this extent, these approaches need to be learned and implemented on models or through training programs with an aim to resolve the problem if it surfaces. A meticulous assessment following the outlined guidelines will enable optimization of patient care over the whole period of the perioperation. The maximum efforts to achieve better results should be encouraged and the comprehensive assessment of all associated potential risk factors for every single patient needs to take care of. Hence, it becomes imperative on the part of anesthesiologists to have a concrete knowledge of the technique with additional capability to handle high-risk patients.

## References

[1] Baraka AS, Taha SK, Aouad MT, El-Khatib MF, Kawkabani N (1999). Pre-oxygenation: comparison of maximal breathing and tidal volume breathing techniques. Anesthesiology.

[2] Benumof JL, Dagg R, Benumof R (1997). Critical hemoglobin desaturation will occur before return to an unparalyzed state following 1 mg/kg intravenous succinylcholine. Anesthesiology.

[3] Weingart SD, Levitan RM (2012). Preoxygenation and prevention of desaturation during emergency airway management. Ann Emerg Med.

[4] Cook TM, Woodall N, Frerk C (2011). Complications of airway management in the UK: results of the fourth national audit project of the Royal College of Anaesthetists and the Difficult Airway Society. Part 1: anaesthesia. Br J Anaesth.

[5] Hamilton WK, Eastwood DW (1955). A study of denitrogenation with some inhalation anesthetic systems. Anesthesiology.

[6] Snow RG, Nunn JF (1959). Induction of anaesthesia in the footdown position for patients with a full stomach. Br J Anaesth.

[7] Kryger MH (1981). Abnormal control of breathing. Pathophysiology of Respiration.

[8] Benumof JL (1999). Preoxygenation: best method for both efficacy and efficiency. Anesthesiology.

[9] Rapaport S, Joannes-Boyau O, Bazin R, Janvier G (2004). Comparison of eight deep breaths and tidal volume breathing preoxygenation techniques in morbid obese patients. Ann Fr Anesth Reanim.

[10] Heller ML, Watson TR Jr (1961). Polarographic study of arterial oxygenation during apnea in man. N Engl J Med.

[11] Campbell IT, Beatty PC (1994). Monitoring preoxygenation. Br J Anaesth.

[12] Lane S, Saunders D, Schofield A, Padmanabhan R, Hildreth A, Laws D (2005). A prospective, randomised controlled trial comparing the efficacy of preoxygenation in the 20 degrees head-up vs supine position. Anaesthesia.

[13] Ramkumar V, Umesh G, Philip FA (2011). Preoxygenation with 20° headup tilt provides longer duration of non-hypoxic apnea than conventional preoxygenation in non-obese healthy adults. J Anesth.

[14] Berry CB, Myles PS (1994). Preoxygenation in healthy volunteers:a graph of oxygen «washin» using end-tidal oxygraphy. Br J Anaesth.

[15] McGowan P, Skinner A (1995). Preoxygenation--the importance of a good face mask seal. Br J Anaesth.

[16] Nimmagadda U, Salem MR, Joseph NJ, Lopez G, Megally M, Lang DJ (2000). Efficacy of preoxygenation with tidal volume breathing. Comparison of breathing systems. Anesthesiology.

[17] Choinière A1, Girard F, Boudreault D, Ruel M, Girard DC (2001). Voluntary hyperventilation before a rapid-sequence induction of anesthesia does not decrease postintubation PaCO2. Anesth Analg.

[18] Russell GN, Smith CL, Snowdon SL, Bryson TH (1987). Pre-oxygenation and the parturient patient. Anaesthesia.

[19] Gambee AM, Hertzka RE, Fisher DM (1987). Preoxygenation techniques:comparison of three minutes and four breaths. Anesth Analg.

[20] Herriger A, Frascarolo P, Spahn DR, Magnusson L (2004). The effect of positive airway pressure during pre-oxygenation and induction of anaesthesia upon duration of non-hypoxic apnoea. Anaesthesia.

[21] McCrory JW, Matthews JN (1990). Comparison of four methods of preoxygenation. Br J Anaesth.

[22] Rooney MJ (1994). Pre-oxygenation:a comparison of two tech¬niques using a Bain system. Anaesthesia.

[23] Wahba WM (1983). Influence of aging on lung function—clinical significance of changes from age twenty. Anesth Analg.

[24] McCarthy G, Elliott P, Mirakhur RK, McLoughlin C (1991). A comparison of different pre-oxygenation techniques in the elderly. Anaesthesia.

[25] Morrison JE Jr, Collier E, Friesen RH, Logan L (1998). Preoxygenation before laryngoscopy in children:how long is enough?. Paediatr Anaesth.

[26] Hardman JG, Wills JS (2006). The development of hypoxaemia during apnoea in children:a computational modelling investigation. Br J Anaesth.

[27] Russell GN, Smith CL, Snowdon SL, Bryson TH (1987). Preoxygenation and the parturient patient. Anaesthesia.

[28] Baraka AS, Hanna MT, Jabbour SI, Nawfal MF, Sibai AA, Yazbeck VG, Khoury NI (1992). Preoxygenation of pregnant and nonpregnant women in the head-up versus supine position. Anesth Analg.

[29] Russel EC, Wrench J, Feast M, Mohammed F (2008). Preoxygenation in pregnancy:the effect of fresh gas flow rates within a circle breathing system. Anaesthesia.

[30] Gunnarsson L, Tokics L, Lundquist H, Brismar B, Strandberg A, Berg B, Hedenstierna G (1991). Chronic obstructive pulmonary disease and anaesthesia:formation of atelectasis and gas exchange impairment. Eur Respir J.

[31] Jense HG, Dubin SA, Silverstein PI, O’Leary-Escolas U (1991). Effect of obesity on safe duration of apnea in anesthetized humans. Anesth Analg.

[32] Dixon BJ, Dixon JB, Carden JR, Burn AJ, Schachter LM, Playfair JM, Laurie CP (2005). Preoxygenation is more effective in the 25 degrees head-up position than in the supine position in severely obese patients:a randomized controlled study. Anesthesiology.

[33] Gil KS, Diemunsch PA (2013). Fiberoptic and flexible endoscopic aided techniques. Benumof’s and Hagberg’s Airway Management.

[34] Lundquist H, Hedenstierna G, Strandberg A, Tokics L, Brismar B (1995). CT-assessment of dependent lung densities in man during general anaesthesia. Acta Radiol.

[35] Hedenstierna G, Edmark L (2010). Mechanisms of atelectasis in the perioperative period. Best Pract Res Clin Anaesthesiol.

[36] Benoît Z, Wicky S, Fischer JF, Frascarolo P, Chapuis C, Spahn DR, Magnusson L (2002). The effect of increased FIO(2) before tracheal extubation on postoperative atelectasis. Anesth Analg.

[37] Cressey DM, Berthoud MC, Reilly CS (2001). Effectiveness of continuous positive airway pressure to enhance pre-oxygenation in morbidly obese women. Anaesthesia.

[38] Daly WJ, Bondurant S (1962). Effects of oxygen breathing on the heart rate, blood pressure, and cardiac index of normal men—resting, with reactive hyperemia, and after atropine. J Clin Invest.

[39] Neill WA (1969). Effects of arterial hypoxemia and hyperoxia on oxygen availability for myocardial metabolism. Patients with and without coronary heart disease. Am J Cardiol.

[40] Rubanyi GM, Vanhoutte PM (1986). Superoxide anions and hyperoxia inactivate endothelium-derived relaxing factor. Am J Physiol.

[41] Kainuma M, Kimura N, Shimada Y (1990). Effect of acute changes in renal arterial blood flow on urine oxygen tension in dogs. Crit Care Med.

[42] Flemming B, Seeliger E, Wronski T, Steer K, Arenz N, Persson PB (2000). Oxygen and renal hemodynamics in the conscious rat. J Am Soc Nephrol.

[43] Jamieson D, Chance B, Cadenas E, Boveris A (1986). The relation of free radical production to hyperoxia. Annu Rev Physiol.

[44] Crystal GJ, Malik G, Yoon SH, Kim SJ (2012). Isoflurane late preconditioning against myocardial stunning is associated with enhanced antioxidant defenses. Acta Anaesthesiol Scand.

[45] Lumb AB (2007). Just a little oxygen to breathe as you go off to sleep... is it always a good idea?. Br J Anaesth.

